# SKILLS FOR PSYCHOLOGICAL RECOVERY DURING AND AFTER DISASTERS TO STRENGTHEN SOCIAL SUPPORT

**DOI:** 10.1093/geroni/igz038.1437

**Published:** 2019-11-08

**Authors:** Lisa M Brown

**Affiliations:** Palo Alto University, Plo Alto, California, United States

## Abstract

The combination of an aging population and a limited number of disaster responders reveals a need for trained laypeople who can implement interventions that facilitate recovery after traumatic events. Skills for Psychological Recovery (SPR) is an intervention that is designed to be implemented after use of Psychological First Aid with people who were exposed to a traumatic stressor. SPR uses a skills-building approach to promote self-efficacy, support resilience, strengthen social networks, and ameliorate the negative impact of traumatic life events. Strengthening social support is beneficial as research clearly demonstrates that destruction of communities and loss of informal social support networks produces long-term psychological distress for older adults. This presentation will demonstrate a SPR social support exercise with attendees, provide an overview of the other modules, and describe modifications for using SPR with older adults. During times of crisis, social support systems are critical to the psychological well-being of older adults.

